# Author Correction: Highly specific and non-invasive imaging of Piezo1-dependent activity across scales using GenEPi

**DOI:** 10.1038/s41467-023-41606-x

**Published:** 2023-09-18

**Authors:** Sine Yaganoglu, Konstantinos Kalyviotis, Christina Vagena-Pantoula, Dörthe Jülich, Benjamin M. Gaub, Maaike Welling, Tatiana Lopes, Dariusz Lachowski, See Swee Tang, Armando Del Rio Hernandez, Victoria Salem, Daniel J. Müller, Scott A. Holley, Julien Vermot, Jian Shi, Nordine Helassa, Katalin Török, Periklis Pantazis

**Affiliations:** 1 Department of Biosystems Science and Engineering, Eidgenössische Technische Hochschule (ETH) Zurich, Basel, Switzerland; 2 Department of Bioengineering, Imperial College London, London, UK; 3 Department of Molecular, Cellular and Developmental Biology, Yale University, New Haven, CT USA; 4 Section of Investigative Medicine, Department of Metabolism, Digestion, and Reproduction, Imperial College London, London, UK; 5 Leeds Institute of Cardiovascular and Metabolic Medicine, LIGHT Laboratories, University of Leeds, Leeds, UK; 6 Molecular and Clinical Sciences Research Institute, St. George’s, University of London, London, UK; 7 Department of Biochemistry, Cell and Systems Biology, Institute of Systems, Molecular and Integrative Biology, University of Liverpool, Liverpool, UK

**Keywords:** Cellular imaging, Calcium signalling, Molecular imaging

Correction to: *Nature Communications* 10.1038/s41467-023-40134-y, published online 19 July 2023

In the original version of this article, the given and family names of Armando Del Rio Hernandez were incorrectly structured. The name was displayed correctly in all versions at the time of publication. In this article the affiliation details for Nordine Helassa were incorrectly given as ‘7. Centre for Cardiovascular Science, Department of Cardiovascular Science and Metabolic Medicine, Institute of Life Course and Medical Sciences, Faculty of Health and Life Sciences, University of Liverpool, Liverpool, UK.’ but should have been ‘7. Department of Biochemistry, Cell and Systems Biology, Institute of Systems, Molecular and Integrative Biology, University of Liverpool, Liverpool, UK.’. The original article has been corrected.

