# Supplementary material for: Highly specific and non-invasive imaging of Piezo1-dependent activity across scales using GenEPi
Source: Nat Commun. 2023 Jul 19;14:4352. doi: 10.1038/s41467-023-40134-y (PMC10356793; doi:10.1038/s41467-023-40134-y)
Supplement: Supplementary file 1 — Supplementary Information [file 41467_2023_40134_MOESM1_ESM.pdf]

Supplementary Information for

Highly specific and non-invasive imaging of Piezo1-dependent activity across scales using GenEPi

Sine Yaganoglu[1]†, Konstantinos Kalyviotis[2]†, Christina Vagena-Pantoula[2], Dörthe Jülich[3], Benjamin M. Gaub[1], Maaike Welling[1,2], Tatiana Lopes[4], Dariusz Lachowski[2], See Swee Tang[2], Armando Del Rio Hernandez[2], Victoria Salem[2], Daniel J. Müller[1], Scott A. Holley[3], Julien Vermot[2], Jian Shi[5], Nordine Helassa[6,7], Katalin Török[6], Periklis Pantazis[1,2*]

†Contributed equally
Correspondence to: p.pantazis@imperial.ac.uk

This file includes:
Supplementary Notes 1 to 3
Supplementary Figures 1 to 22
Supplementary Tables 1 to 3
Supplementary Movies 1 to 4

Supplementary Note 1-Endoplasmic reticulum (ER) and plasma membrane localization of GenEPi/loss-of-function GenEPi-S217L and validation of whole cell fluorescence measurement.

Endogenous Piezo1 localizes to the plasma membrane as well as to the ER. However, when fused to fluorescent proteins, membrane ion channels can show poor membrane localization thereby reducing the resolution of the reporter signal[1]. Therefore, we investigated whether the fusion of GCaMPs to Piezo1 would affect its cellular localization. First, we confirmed plasma membrane localization of GenEPi by introducing a live membrane stain, Wheat Germ Agglutinin (WGA)-conjugated Alexa Fluor 594 (magenta), to GenEPi-transfected HEK 293T cells (green). The fluorescent signal of GenEPi overlapped with the live membrane stain, indicating correct plasma membrane localization for GenEPi (**Supplementary Note 1-Fig. 1a-c**). GCaMP-G4 alone however was excluded from the cell membrane (**Supplementary Note 1-Fig. 1a-c**). We next investigated whether the GenEPi is localized in the ER. GenEPi (green) expressing HEK 293T cells were labeled with BODIPY TR Gilbenclamide (magenta), a live stain for the ER. Intracellular GenEPi signal overlapped with the BODIPY TR Gilbenclamide staining, confirming ER localization of the reporter (**Fig. 1**).

Loss-of-function missense mutations of Piezo1 have been linked to several diseases, such as generalized lymphatic dysplasia[10,11], and bicuspid aortic valve [91]. Recently, the loss-of-function S217L (Serine 217 to Leucine) missense mutation of Piezo1 has been shown to exhibit reduced plasma membrane trafficking, reduced stability and higher ubiquitination compared to wt-Piezo1[26]. To further test the functionality of GenEPi and confirm that it can recapitulate Piezo1 disease conditions, we used site-directed mutagenesis to generate a GenEPi-S217L variant and tested whether the GenEPi-S217L mutant exhibits similar subcellular localization. Non-mutated GenEPi localizes both in the plasma membrane and the ER reflecting the subcellular localization of wt-Piezo1 (**Supplementary Note 1-Fig. 1d**), whereas GenEPi-S217L co-localizes almost exclusively with the ER tracker (**Supplementary Note 1-Fig. 1d-e**) like Piezo1-S217L[26]. In contrast to non-mutated GenEPi, GenEPi-S217L does not respond to treatment with 10μM of Yoda1 (**Supplementary Note 1-Fig. 1f**). Interestingly, GenEPi-S217L shows a significant decrease in its fluorescence upon agonist stimulation. Overall, we demonstrate that GenEPi reflects the subcellular localization of wt-Piezo1 and can recapitulate Piezo1 disease conditions which are linked to loss-of-function mutations.

To confirm that our whole cell measurement of GenEPi fluorescence for various experiments reflects the changes in the membrane GenEPi signals with little interference from the ER, we compared plasma membrane F/F_0 measurements to whole cell measurements in a subset of cells. To isolate membrane signals, we stained GenEPi-expressing cells with WGA-Alexa Fluor 594, a live cell stain for the cell membrane (magenta). We exposed the cells to 10 dyn/cm^2 fluid shear stress and recorded the response of GenEPi simultaneously with the static membrane staining. We used the WGA-Alexa Fluor 594 channel to generate a mask of the cell membrane (**Supplementary Note 1-Fig. 1g**). This mask was applied to GenEPi signals, to extract the cell membrane-specific fluorescence signal of GenEPi. We compared the F/F_0 measurements from the membrane to that of the whole cell response of the same cells and found no significant difference (**Supplementary Note 1-Fig. 1h**). Hence, whole cell responses accurately reflect cell membrane GenEPi dynamics.

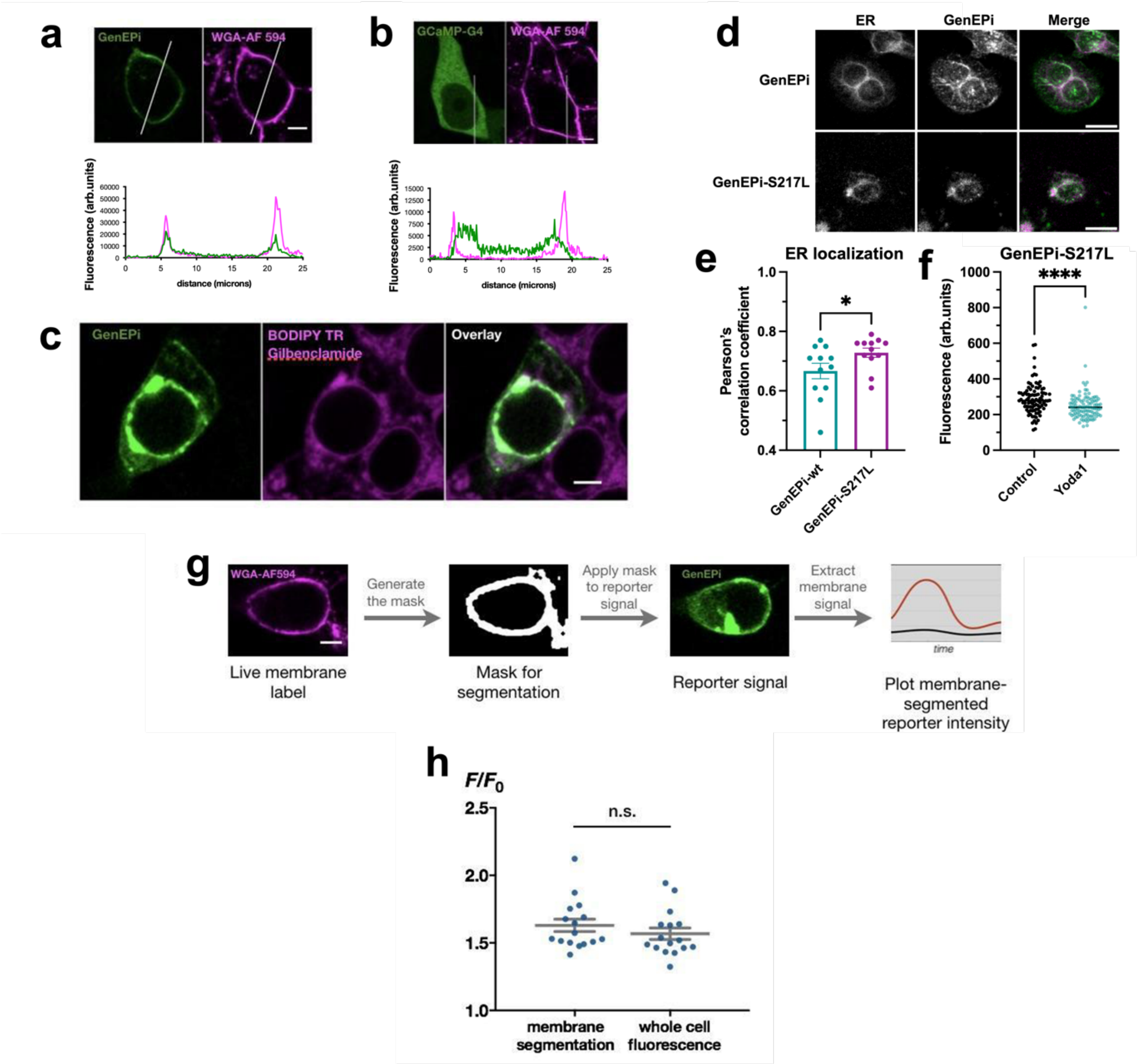

Supplementary Note 1-Fig. 1. Intracellular localization of GenEPi and validation of whole cell fluorescence measurement. Fluorescence signal intensity profiles of a cross-section through a representative HEK 293T cell expressing (**a**) GenEPi (green) or (**b**) GCaMP-G4 stained with Wheat Germ Agglutinin conjugated Alexa Fluor 594 (magenta). (**c**) GenEPi expressing (green) HEK 293T cell stained with BODIPY TR Gilbenclamide (magenta), a live stain for ER. (**d**) Representative images of HeLa cells expressing GenEPi or GenEPi-S217L stained with ER-tracker to visualize ER and anti-GFP to visualize GenEPi. Scale bar, 20µM. (e) Summary data showing the overlap of GenEPi (wt- or S217L) and the ER signal indicated by Pearson's correlation coefficient. (n=12 for each). Data are presented as means ± SEM. Mann-Whitney test. (**f**) Response of GenEPi-S217L expressing HeLa cells to 10µM Yoda1 (n=104) or DMSO (control) (n=92). Data are presented as means ± SEM. Mann-Whitney test. (**g**) Segmentation procedure to obtain plasma membrane masks: live plasma membrane stain (magenta) is used as a mask to extract membrane signals from GenEPi expressing (green) cells. Obtained signals are then plotted over time. (**h**) Comparison of segmented membrane F/F_0 measurements to whole cell measurements. n=16 cells, Two-tailed Mann-Whitney test, n.s.= p>0.05. Data are presented as means ± SEM., data from three independent experiments. (**a-d**) Scale bar, 5 µm. Source data are provided as a Source Data file.

Supplementary Note 2-Plasma membrane localization of GCaMP-G4 does not confer functional specificity.

As GenEPi responded specifically to mechanical signals and was unresponsive to cytosolic calcium fluctuations, we wondered whether the demonstrated functional specificity could be also achieved by simply targeting GCaMP-G4 to the plasma membrane. To this end, we attached the membrane targeting sequence of the protein tyrosine kinase Lck to the N-terminus of the calcium indicator GCaMP-G4 (Lck-GCaMP-G4). We then exposed Lck-GCaMP-G4 and Piezo1 co-transfected cells to fluid shear stress and ionomycin, and compared the responses of Lck-GCaMP-G4 and GenEPi. If simply being located to the membrane was sufficient to give i) a strong response to fluid shear stress and ii) a negligible response to ionomycin, Lck-GCaMP-G4 would be expected to behave similarly to GenEPi. In contrast, the response of Lck-GCaMP-G4 to fluid shear stress was significantly lower than that of GenEPi, while it showed a pronounced response to ionomycin (**Supplementary Note 2-Fig. 1**).

Hence, membrane localization alone is not sufficient to acquire functional specificity to mechanical stimuli. Discrete high Ca^{2+} concentrations in the immediate vicinity of the Piezo1 channel opening could be causing these differences, such as the previously reported Ca^{2+} microdomains[17]. Indeed, the fact that all Piezo1- fused variants gave lower responses to ionomycin than cytosolic GCaMPs (**Fig. 1C**) corroborates this hypothesis.

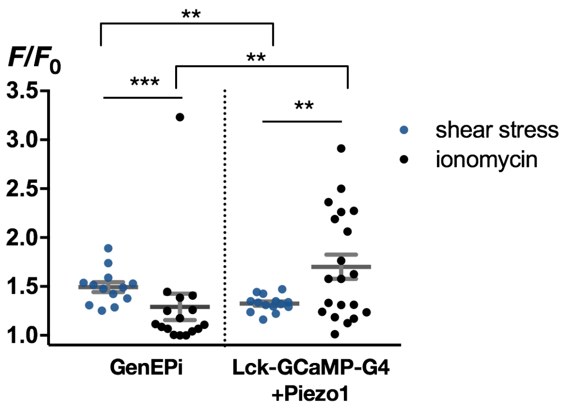

Supplementary Note 2-Fig. 1. Plasma membrane localization of GCaMP-G4 alone does not confer functional specificity. Responses of GenEPi or Lck-GCaMP-G4 with Piezo1 expressing HEK293T cells to 10 dyne/cm^2 fluid shear stress (GenEPi: n=13, Lck-GCaMP-G4+Piezo1: n=15) or 1 µM ionomycin (GenEPi: n=15, Lck-GCaMP-G4+Piezo1: n=20). GenEPi shear vs. GenEPi ionomycin and GenEPi vs Lck-GCaMP-G4 ionomycin response, Two-tailed Mann-Whitney test, **= p<0.01, ***= p<0.001. Lck-G4 shear vs. GenEPi shear response and Lck-G4 shear vs. Lck-G4 ionomycin two-tailed Welch's t-test, **= p<0.01. are means +/- SEM, data from 4 independent experiments. Source data are provided as a Source Data file.

Supplementary Note 3-Conversion of force values to pressure values.

To compare the force threshold values with published pressure values we calculated the pressure resulting from mechanical stimulations pushing a 5 µm diameter bead onto the cell body. The maximum contact area *A* between bead and cell is given as half sphere surface of the bead:

$$A = 2\pi\, r^2 \qquad \text{Equation S1}$$

with r = 2.5 µm, the area A is:

$$A = 2\pi(2.5\ \mu m)^2 = 39.3\ \mu m^2$$

Accordingly, the pressure P is given as:

$$P = \frac{F}{A} \qquad \text{Equation S2}$$

with F being the force pushing the bead onto the cell. The pressure P corresponding to the compressing force was calculated the following way. For 100 nN, P is:

$$P = \frac{100 * 10^{-9}\ kg\ m\ s^{-2}}{39.3 * 10^{-12}\ m^2} = 2.55 * 10^3 kg\ m^{-1}\ s^{-2} = 2.55\ kPa$$

$1\ kPa = 7.5\ mm\ Hg$, therefore:

$$2.55\ kPa = 19.13\ mm\ Hg$$

Using these equations, we can obtain the following pressure values for given compressing forces:

100nN = 2.6 kPa or 19.1 mmHg; 200 nN = 5.1 kPa or 38.3 mmHg; 300 nN = 7.6 kPa or 57.4 mmHg; 400 nN = 10.2 kPa or 76.5 mmHg;

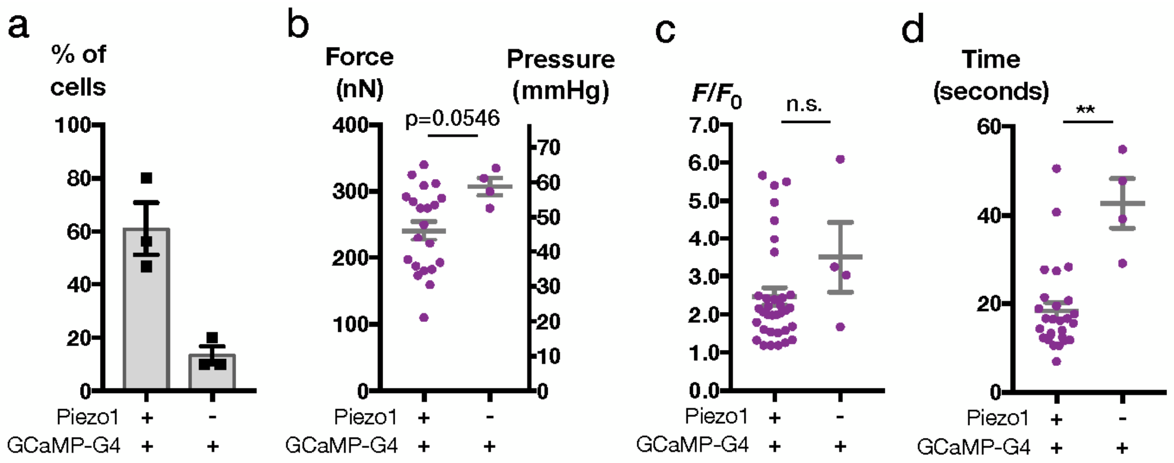

Supplementary Fig. 1. Response of HEK 293T cells to compressive forces is dependent on Piezo1 overexpression. (a) Percentage of cells transfected with cytosolic GCaMP-G4 alone or co-transfected with human Piezo1, that responded to mechanical stimulation using AFM in each experiment (black squares). n=3 experiments. In total 41 cells tested for GCaMP-G4 co-expression with Piezo1 and 30 cells tested for GCaMP-G4 expression alone. Data are presented as means ± SEM. Note that stimulated HEK 293T cells transfected only with cytosolic GCaMP-G4 rarely responded to compressive forces. (b) Threshold forces and pressures for cells transfected with GCaMP-G4 alone (n=4 cells) or co-transfected with human Piezo1 (n=21 cells). Two-tailed unpaired t-test, p=0.0546. (c) Amplitudes of optical calcium responses or cells transfected with GCaMP-G4 alone (n=4 cells) or co-transfected with human Piezo1 (n=34 cells). Two-tailed Mann-Whitney test, **=p<0.01. (d) Durations of optical calcium responses of cells transfected with GCaMP-G4 alone (n=4 cells) or co-transfected with human Piezo1 (n=27 cells). Two-tailed Mann-Whitney test, n.s.= p>0.05. Purple dots are single cells, data are presented as means ± SEM. Data from three independent experiments. Note that the threshold force values were considerably higher and the peak duration was substantially longer for responsive cells that were transfected only with cytosolic GCaMP-G4 than for cells with Piezo1 overexpression. Source data are provided as a Source Data file.

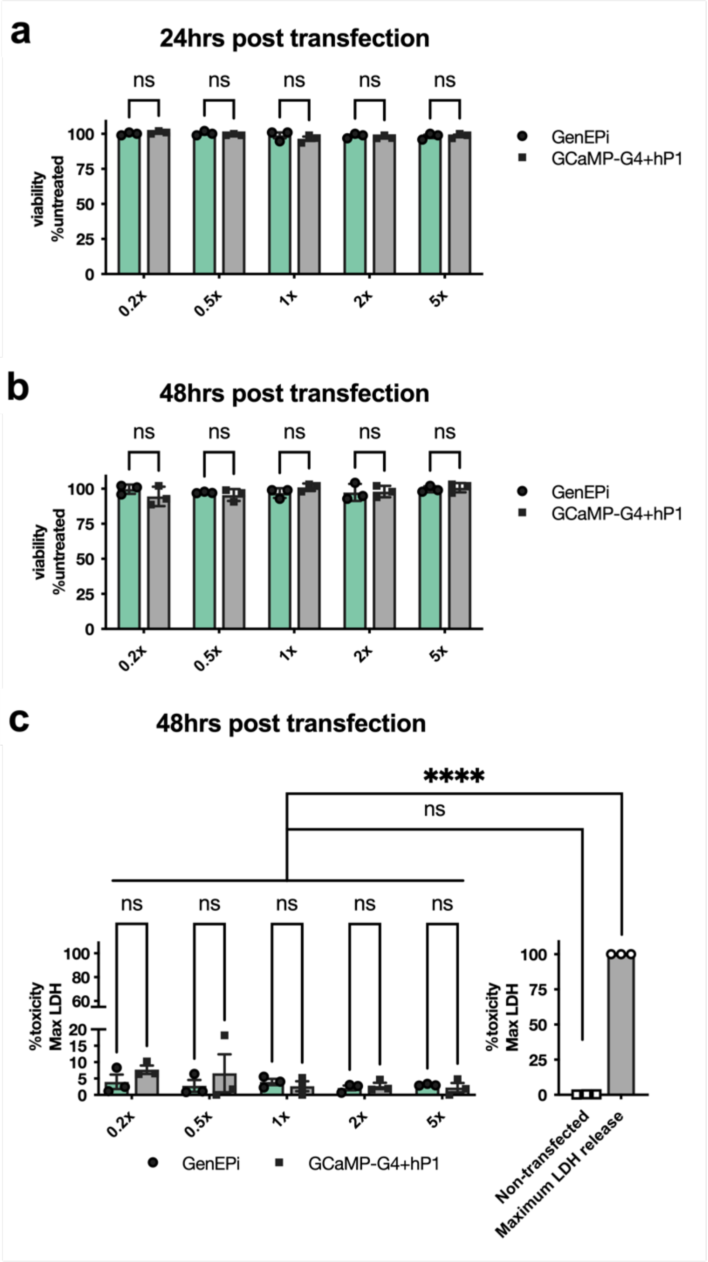

Supplementary Fig. 2. GenEPi expression does not affect cell viability and does not induce toxicity. (a) The viability of HEK 293T cells expressing either GenEPi or GCaMP-G4 and Piezo1 in the normal transfection dose (1x), as well as in lower (0.2x and 0.5x) and higher transfection doses (2x and 5x) is not affected at 24 hours post transfection and (b) 48 hours post transfection. Two-tailed unpaired t-test. (c) Assessment of the cell toxicity via LDH release at 48 hours post transfection show that transfection of various doses of GenEPi or GCaMP-G4 and Piezo1 induces negligible amounts of cell toxicity. Two-tailed unpaired t-test. For all experiments (n=3 wells), error bar= SEM, data from three independent experiments. Source data are provided as a Source Data file.

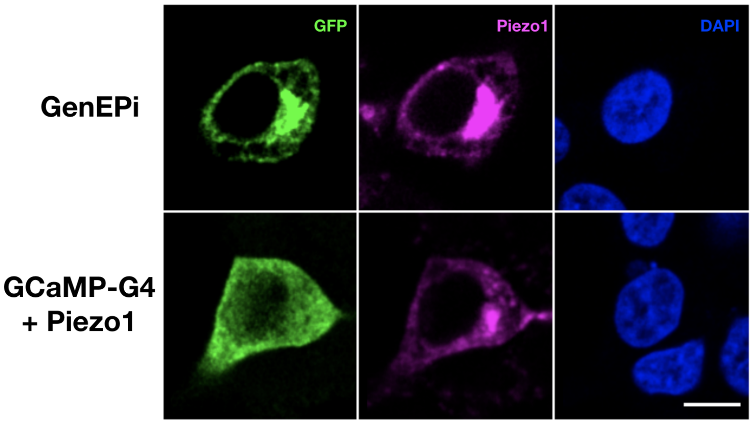

Supplementary Fig. 3. Comparable subcellular localization of GenEPi and wild-type Piezo1. GenEPi (green) expressing (upper panel) or GCaMP-G4 and Piezo1 co-expressing (lower panel) HEK 293T cell stained with antibody against GFP which binds to GCaMP (green), Piezo1 (magenta), and DAPI (blue). Scale bar, 10 μm.

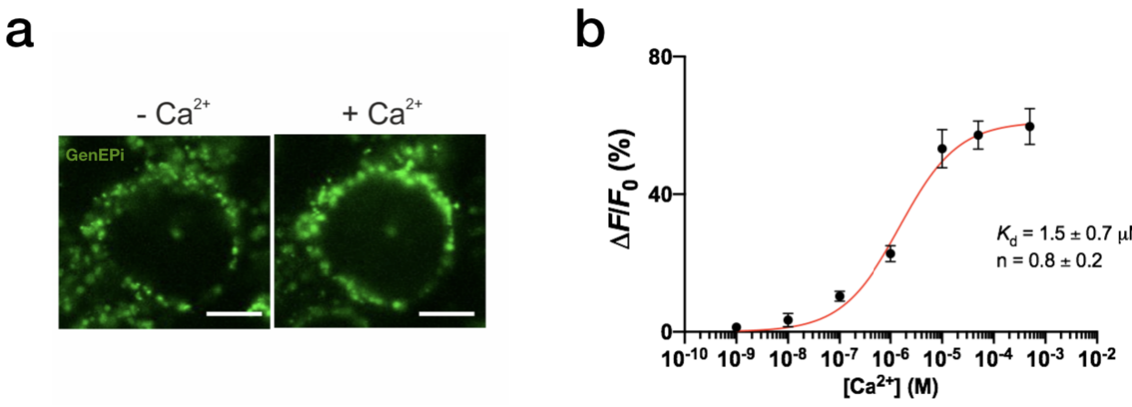

Supplementary Fig. 4. *In* situ Ca^{2+} titration shows that GenEPi retains low affinity for Ca^{2+}. (a) Representative confocal images of HeLa cells expressing GenEPi showing plasma membrane localization and fluorescence increase in the presence of 100 µM Ca^{2+}. Scale bar, 10 µm. (b) *In situ* Ca^{2+} titration of GenEPi. Ca^{2+}-dependent changes in fluorescence intensities were fitted to the Hill equation to estimate K_d (dissociation constant) and n (Hill coefficient). Fluorescence dynamic range $(F_{max}-F_0)/F_0$ or $\Delta F/F_0$ in percentages was expressed as mean ± SEM (n=12 to 42 cells from at least three independent experiments). Source data are provided as a Source Data file.

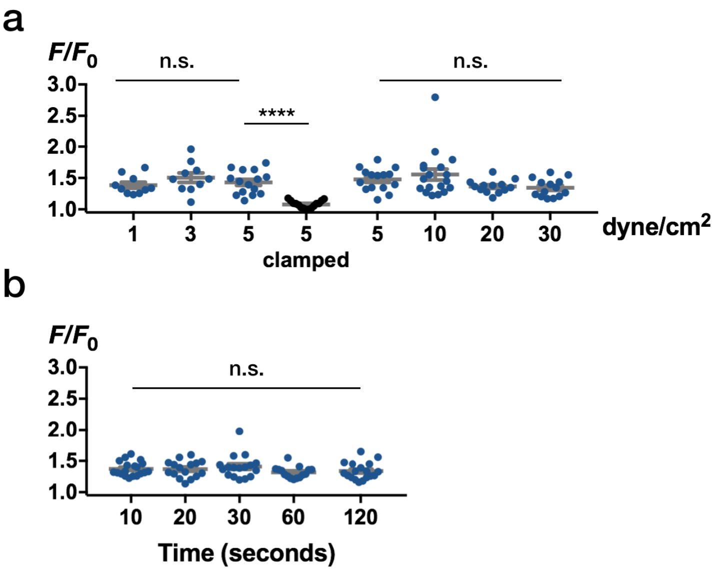

Supplementary Fig. 5. Level or duration of applied fluid shear stress does not affect the response amplitude of GenEPi. (a) F/F_0 response of GenEPi transfected HEK 293T cells exposed for 60 seconds to different levels of shear stress (1-30 dyne/cm^2), along with a control at 5 dyne/cm^2 with preventing liquid flow by clamping the tubing. To compare 1 dyn/cm^2 (n=10), 3 dyne/cm^2 (n=10), and 5 dyn/cm^2 (n=15), One-way ANOVA test was used. To compare 5 dyne/cm^2 clamped vs. free flow, Welch's t-test was used. To compare 5 dyne/cm^2 (n=15), 10 dyne/cm^2 (n=18), 20 dyne/cm^2 (n=13), and 30 dyne/cm^2 (n=14), two-tailed Kruskal-Wallis test. ****=p<0.0001, n.s.=p>0.05. Data are presented as means ± SEM., data from three independent experiments. (b) F/F_0 response of GenEPi transfected HEK 293T cells exposed to 10 dyn/cm^2 shear stress for 10 seconds (n=19), 20 seconds (n=16), 30 seconds (n=17), 60 seconds (n=15), and 120 seconds (n=18). Kruskal-Wallis test, n.s.= p>0.05. Data are presented as means ± SEM., data from four independent experiments. Source data are provided as a Source Data file.

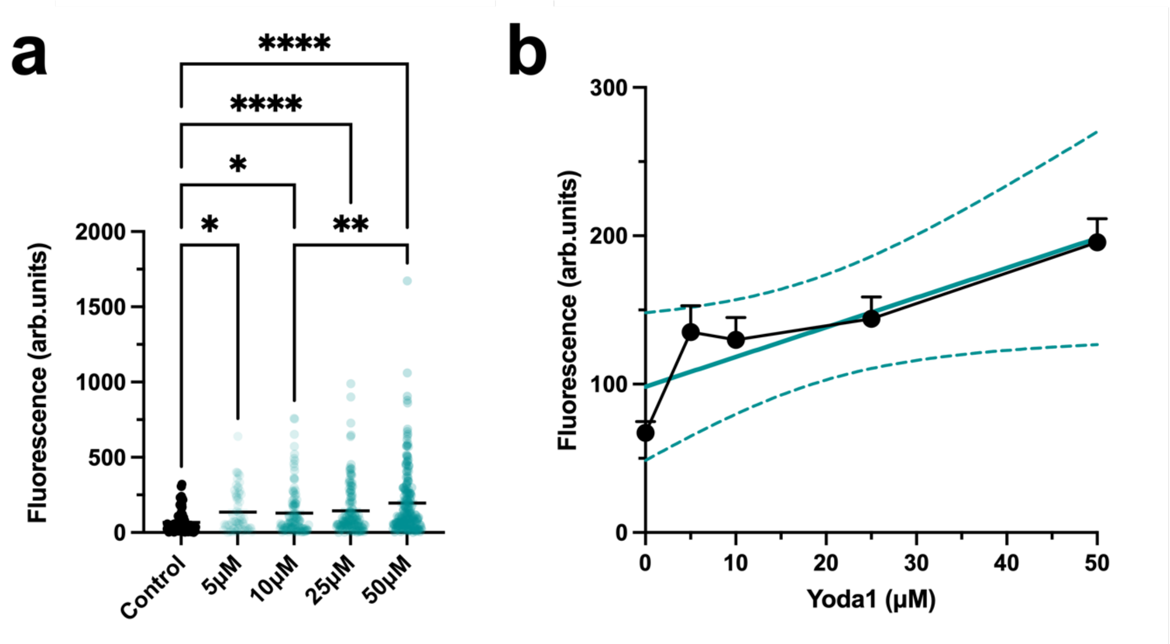

Supplementary Fig. 6. GenEPi responds to the Piezo1-specific agonist Yoda1 in a dose-dependent manner. (a) Response of GenEPi expressing HeLa cells to a range of Yoda1 concentrations: 5µM (n=62), 10µM (n=115), 25µM (n=137) and 50µM (n=197) or DMSO (control) (n=100). Kruskal-Wallis test. (b) GenEPi optical response to Yoda1 strongly correlates with the concentration of Yoda1. Simple linear regression model (solid green line) fitted on the means of each condition in (a). 95% confidence interval (CI) shown as dashed lines, error bars: SEM. R^2=0.7798. Source data are provided as a Source Data file.

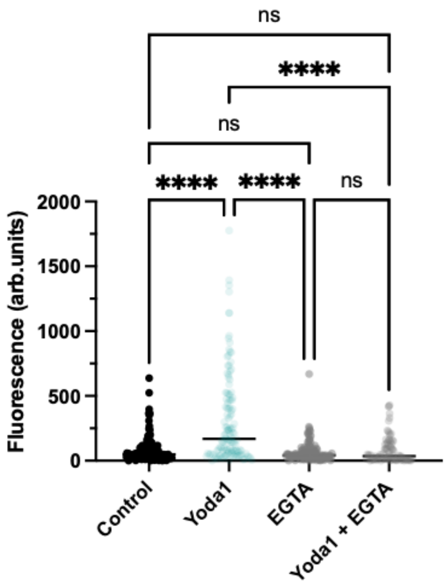

Supplementary Fig. 7. GenEPi shows Piezo1-specific response that critically depends on extracellular calcium influx which can be abolished by extracellular calcium chelation. Response of GenEPi expressing HeLa cells to 10µM Yoda1 (n=124), 2mM EGTA (n=129) and 2mM EGTA and 10µM Yoda1 (n=82) or DMSO (control) (n=134). Data are presented as means ± SEM. Kruskal-Wallis test. Source data are provided as a Source Data file.

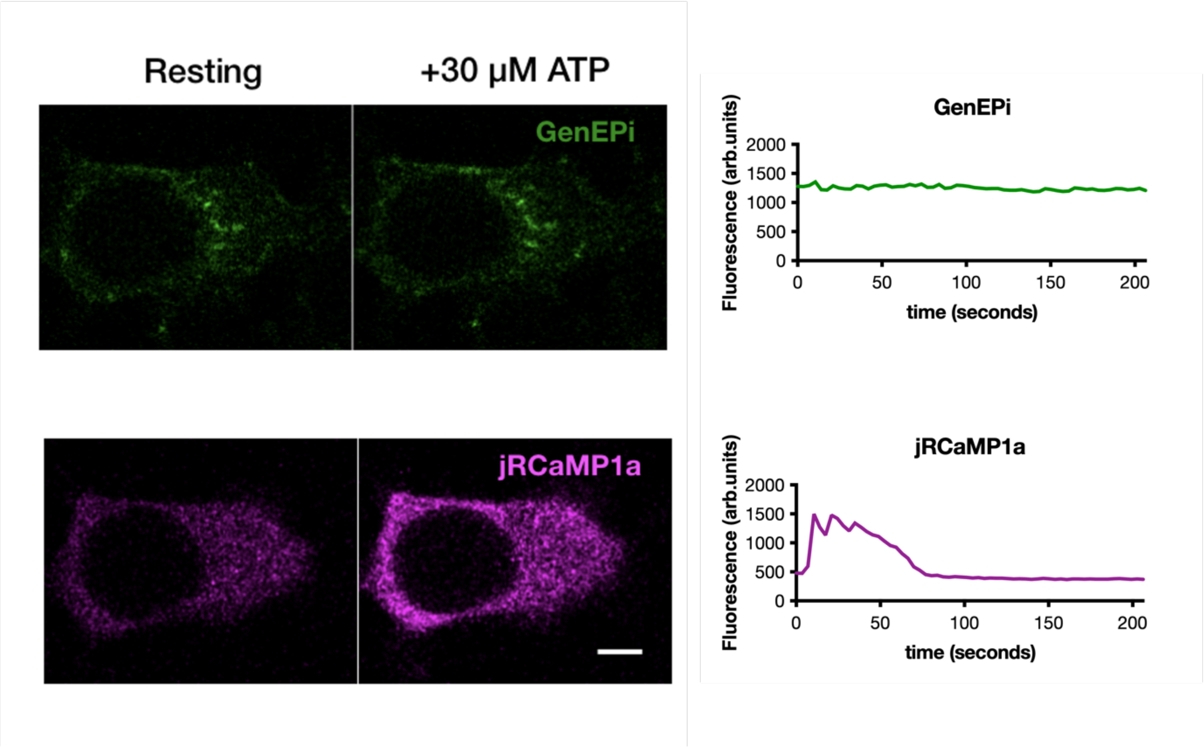

Supplementary Fig. 8. GenEPi does not respond to ATP-induced intracellular calcium fluctuations. Representative images (left panel) of HEK293T cell expressing both GenEPi (green) and jRCaMP1a (magenta) stimulated with 30 µM ATP, along with a fluorescence intensity plotted over time after addition of ATP (right panel). Scale bar, 5 µm. Source data are provided as a Source Data file.

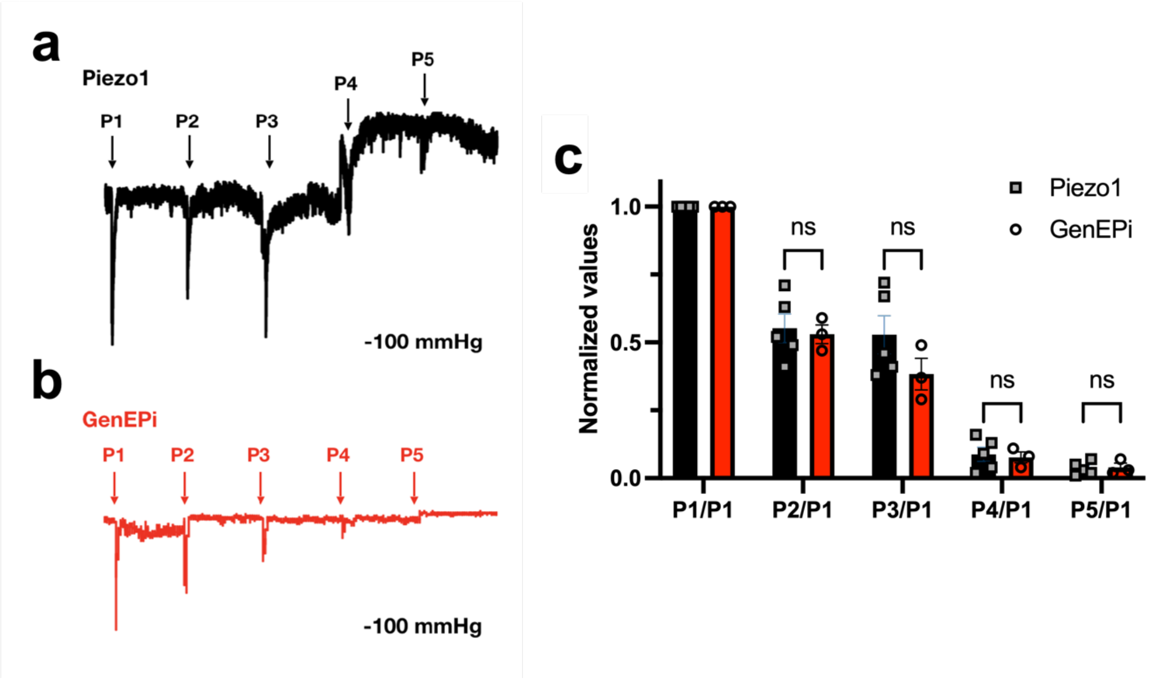

Supplementary Fig. 9. The mechanosensitive response to repetitive stimulation of Piezo1 within GenEPi is similar to wild-type Piezo1. Representative traces of (a) wild-type Piezo1 and (b) GenEPi stimulated by repetitive negative pressure of -100 mmHg every 5 seconds at -80 mV with cell-attached recording configuration. (c) The peak current evoked by each negative pressure was normalized to the first one induced by the same pressure. Both wild-type Piezo1 and GenEPi show a similar response to repetitive negative pressure. Note that 5 out of 9 recordings in wild-type Piezo1 and 3 out of 9 recordings in GenEPi showed responses like the representative traces. Other recordings showed that no response was evoked by the repetitive stimuli of negative pressure after the first one. Data are presented as means ± SEM. Two-tailed unpaired t-test, $p=0.78>0.05$ for P2/P1, $p=0.21>0.05$ for P3/P1, $p=0.78>0.05$ for P4/P1 and $p=0.83>0.05$ for P5/P1. Source data are provided as a Source Data file.

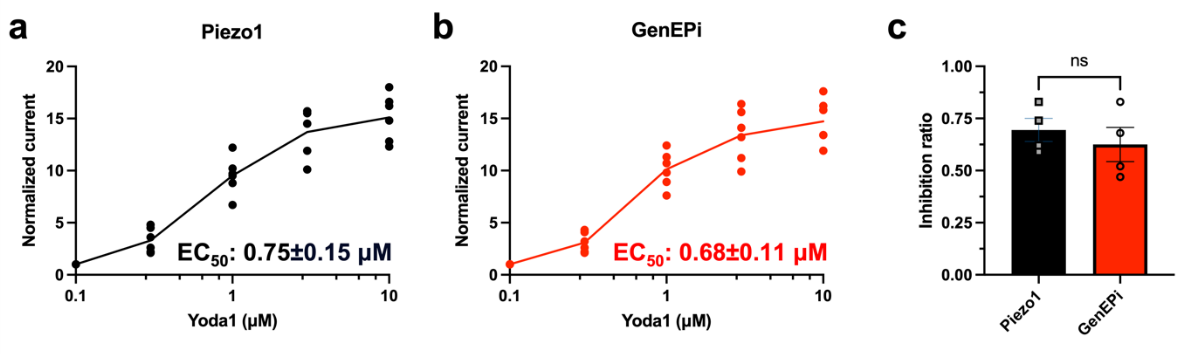

Supplementary Fig. 10. The pharmacological property of Piezo1 within GenEPi does not differ from wild-type Piezo1. (a) Yoda1 dose-dependent curve of wild-type Piezo1 currents shows that the EC_{50} value stays at 0.75±0.15 μM (n=6 cells from 6 experiments). (b) Dose-response curve also gives a similar EC_{50} value for GenEPi. The value is 0.68±0.11 μM (n=6 cells from 6 experiments). (c) Ratio inhibition of 10 μM ruthenium red to both wild-type Piezo and GenEPi currents induced by 10 μM Yoda1 is similar. Inhibition ratios of 10 μM ruthenium red to wild-type Piezo1 (0.70±0.06; n=4), and GenEPi (0.63±0.08; n=4) currents induced by 10 μM Yoda1 are similar without significant difference. Data are presented as means ± SEM. Two-tailed unpaired t-test, n.s.=p>0.05. The whole-cell currents were recorded in the absence of mechanical stimulation. Source data are provided as a Source Data file.

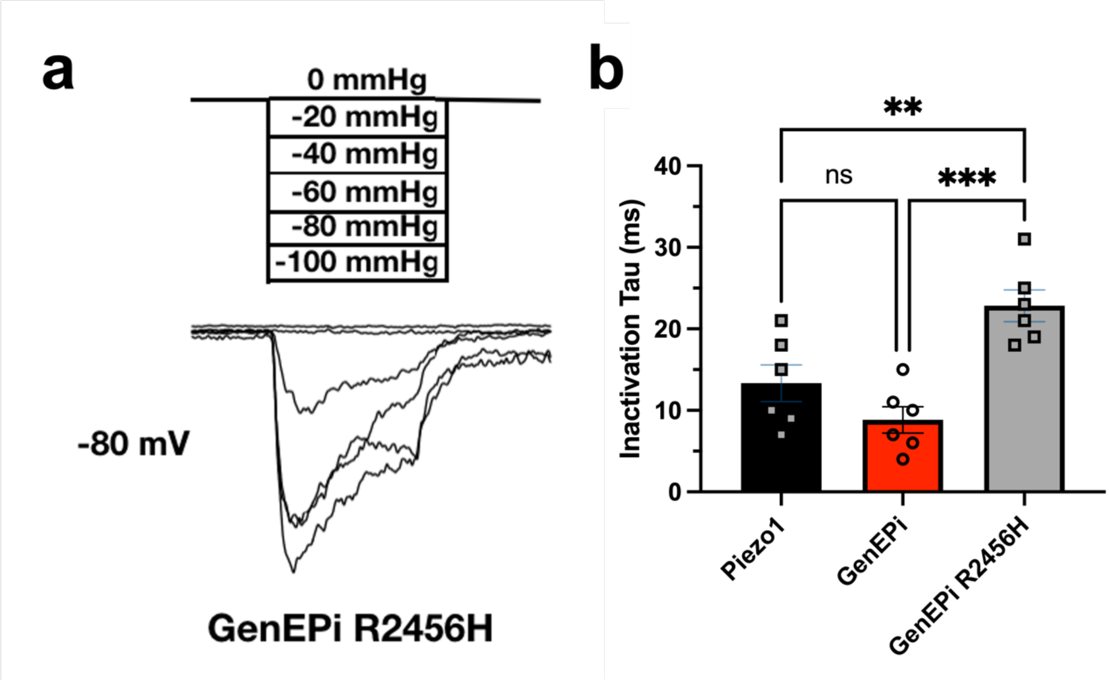

Supplementary Fig. 11. The GenEPi R2456H mutant displays a delayed inactivation phenotype. (a) Representative traces of GenEPi R2456H mutant currents induced by the same negative pressures as the ones used in **Fig. 2L** for wild-type Piezo1 and GenEPi (-0, -20, -40, -60, -80, -100 mmHg) at -80 mV with cell-attached recording configuration. The currents have a delayed inactivation kinetics. (b) The inactivation time (Tau) of GenEPi R2456H mutant is significantly longer than the inactivation time of either wild-type Piezo1 or GenEPi (n= 6 cells for 6 experiments each) at -60 mmHg. Data are presented as means ± SEM. Ordinary one-way ANOVA test, **p=0.009<0.01 between Piezo1 and GenEPi R2456H, ***p=0.0002<0.001 between Piezo1 and GenEPi. Source data are provided as a Source Data file.

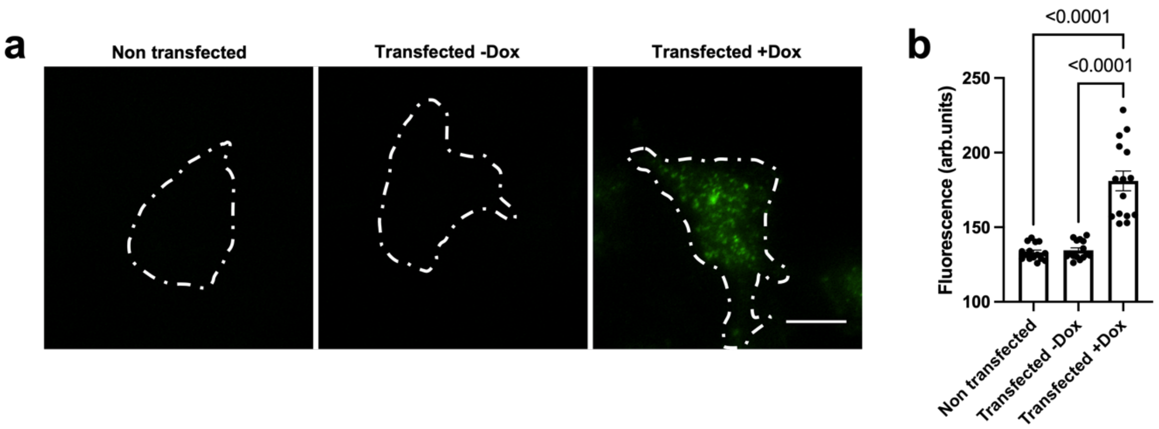

Supplementary Fig. 12. Doxycycline induction of GenEPi expression in the membrane of XLGenEPi transfected HEK293T cells used for TIRFM imaging. (a) Representative TIRFM images of non-transfected, XLGenEPi transfected without doxycycline induction and XLGenEPi transfected with doxycycline induction HEK293T cells. Scale bar, 10µm. (b) Doxycycline induction leads to increased GenEPi expression and SNR. Whole cell fluorescence of n=15 cells, error bar: SEM. Ordinary one-way ANOVA test. Source data are provided as a Source Data file.

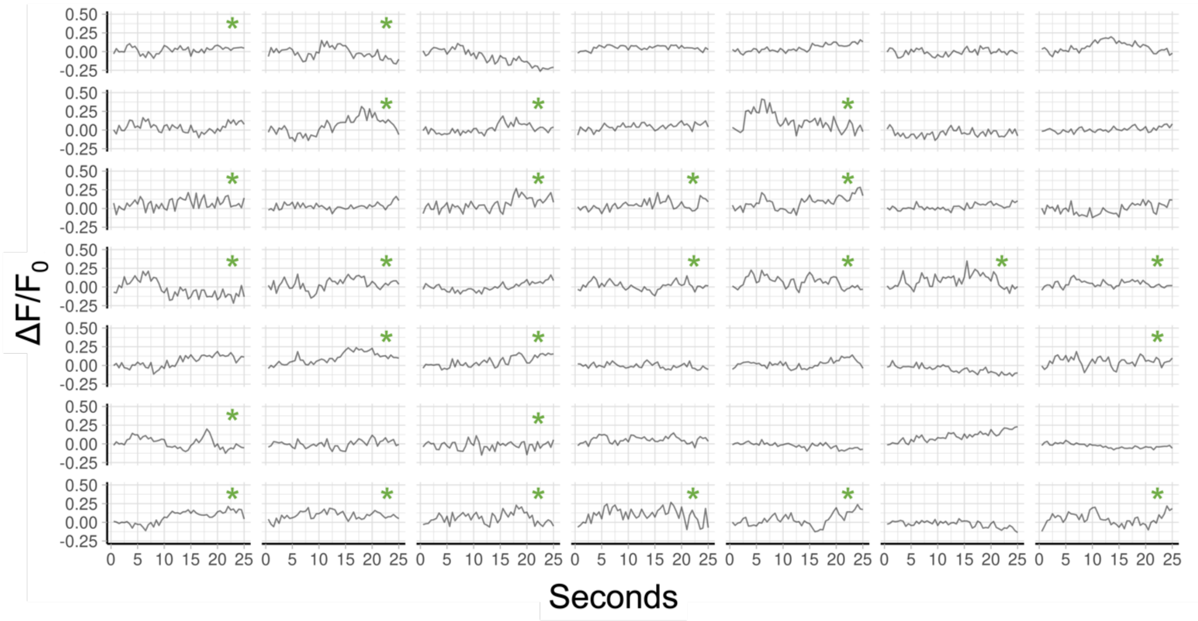

Supplementary Fig. 13. GenEPi responses on the ventral surface of HEK293T cells. GenEPi responses ($\Delta F/F_0$) of 49 individual clusters over the ventral surface of the HEK293T cell presented in Fig. 3i captured using TIRFM imaging. Clusters which showed distinguishable responses are labelled with green stars. Data were plotted in PlotTwist (Shiny apps). Source data are provided as a Source Data file.

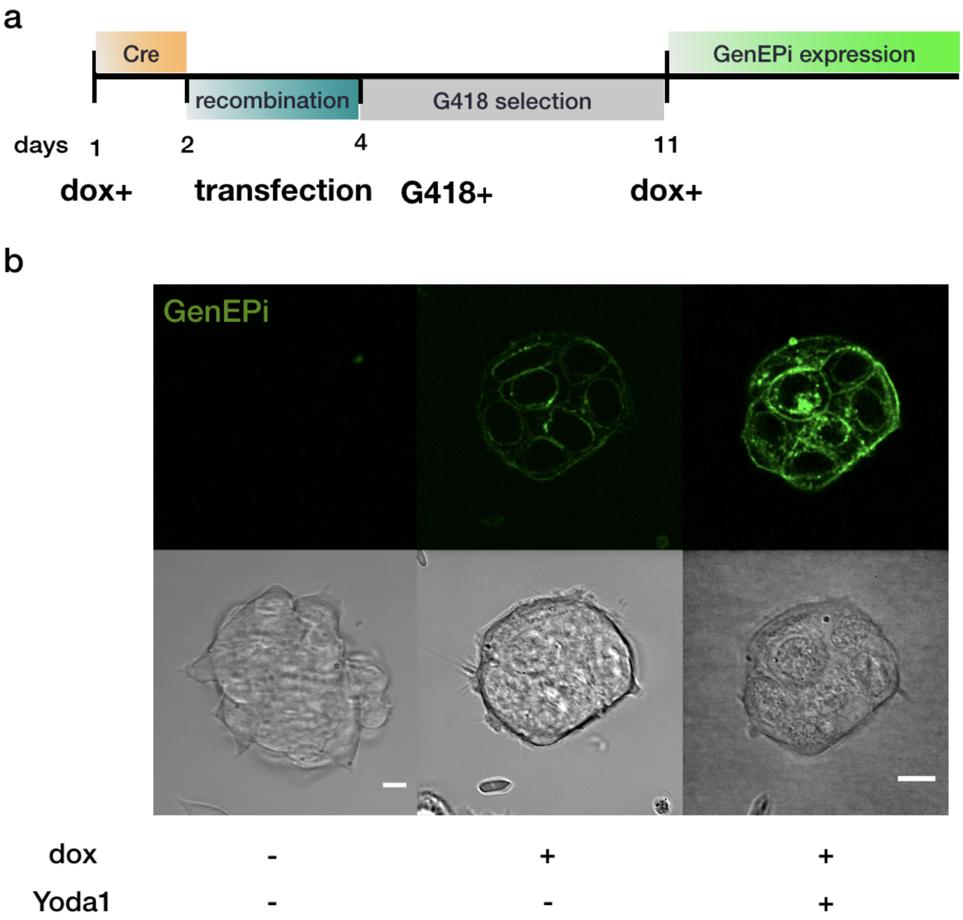

Supplementary Fig. 14. Generation of doxycycline-inducible GenEPi mESC line and its response to Yoda1. (a) Overview of the protocol to generate the doxycycline-inducible GenEPi mESC line. HPRT locus inducible cassette exchange strategy involves the induction of Cre by doxycycline (dox) in transiently transfected cells allowing recombination. Positive clones were selected using G418 and transgene expression was induced using doxycycline. (b) Representative microscopy images of Dox-inducible GenEPi mESC colony without dox treatment (first column), dox-induced GenEPi mESC colony before (second column) and after addition of the Piezo1 channel activator Yoda1 (third column). Scale bar, 10 µm.

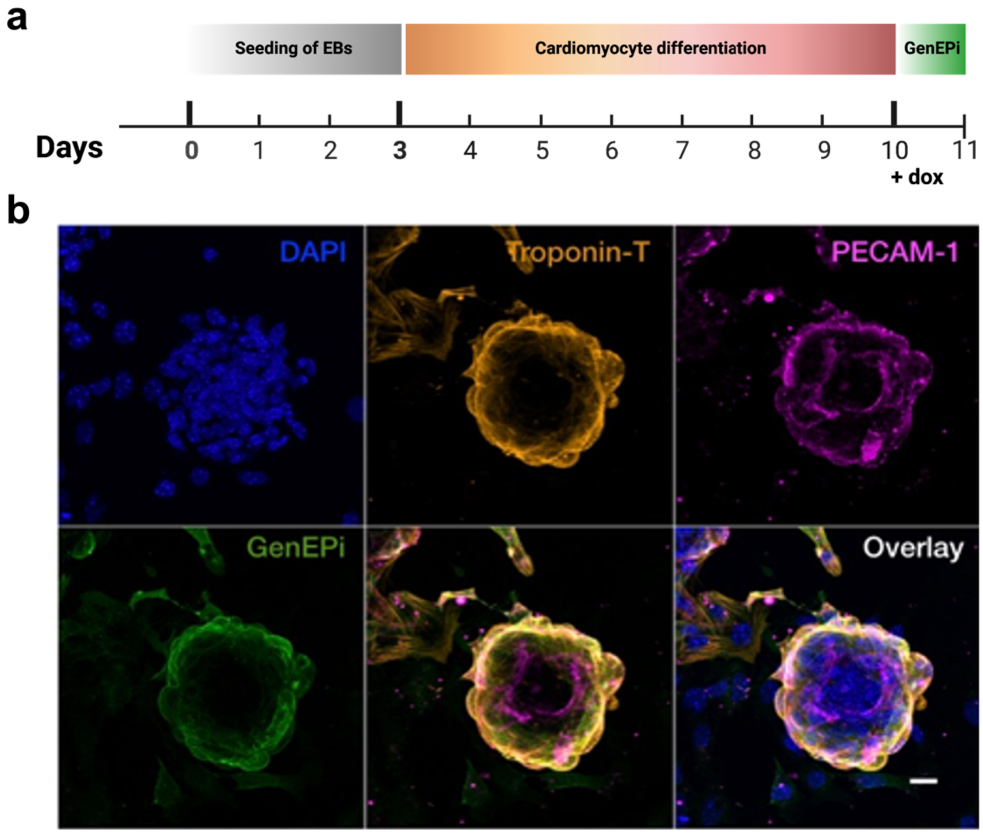

Supplementary Fig. 15. Cardiomyocyte differentiation of doxycycline-inducible GenEPi mESC cell line. (a) Time course for cardiomyocyte differentiation, starting with seeding embryoid bodies followed by incubation with Wnt/β-catenin inhibitor XAV939. Within 10 days, small microtissues consisting of beating cardiomyocytes are obtained and GenEPi expression is detected in the presence of dox. (b) A representative GenEPi expressing beating patch fixed and stained with anti-GFP antibody (green) for GenEPi, an anti-Troponin T antibody (orange) for cardiomyocytes, and PECAM-1 (purple) for endothelial cells, and DAPI (blue). Scale bar, 20 µm.

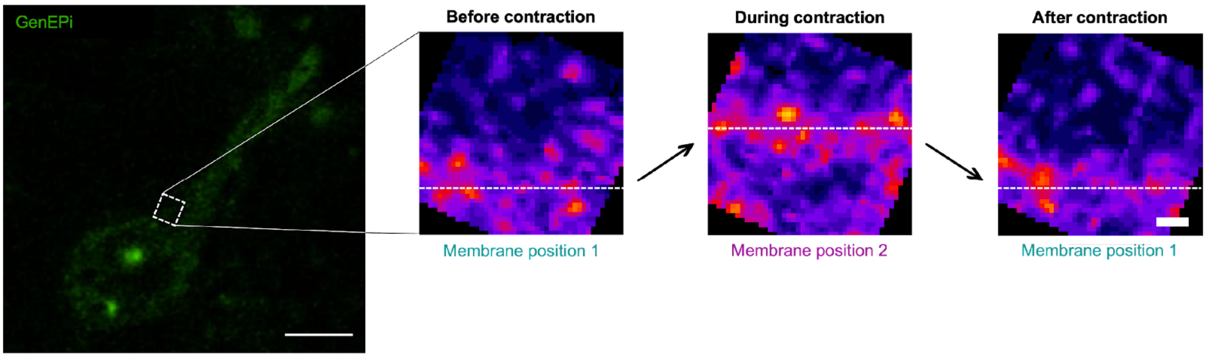

Supplementary Fig. 16. Local activation of GenEPi is caused by membrane pulling during contraction. GenEPi is activated in response to the contraction of autonomously beating cardiomyocytes which cause local membrane displacement. In the "Before contraction" and "After contraction" frames the membrane is in "Membrane position 1", while during the contraction the membrane of the GenEPi expressing cell is displaced upwards by 1 µm to "Membrane position 2". Scale bars, 5 µm (left image) and µm (right panel).

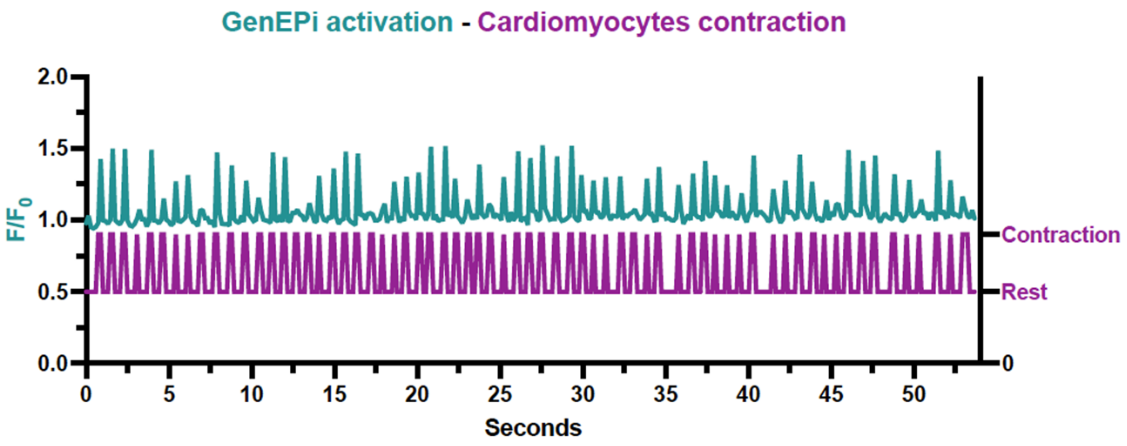

Supplementary Fig. 17. GenEPi activation resolves cardiomyocyte contraction-triggered mechanical stimulation with high temporal resolution. Representative fluorescence intensity (F/F_0) profile (teal line) of GenEPi activation in response to cardiomyocyte contraction (purple line). Cardiomyocyte contraction was captured by changes in the intensity profile of the brightfield image due to cell movement. Data were transformed to a binary response with a minimum of 0.5 (rest) and maximum of 0.9 (contraction) for illustration purposes. GenEPi is activated in response to mechanical stimuli and membrane deformations due to the contraction of autonomously beating cardiomyocytes. Source data are provided as a Source Data file.

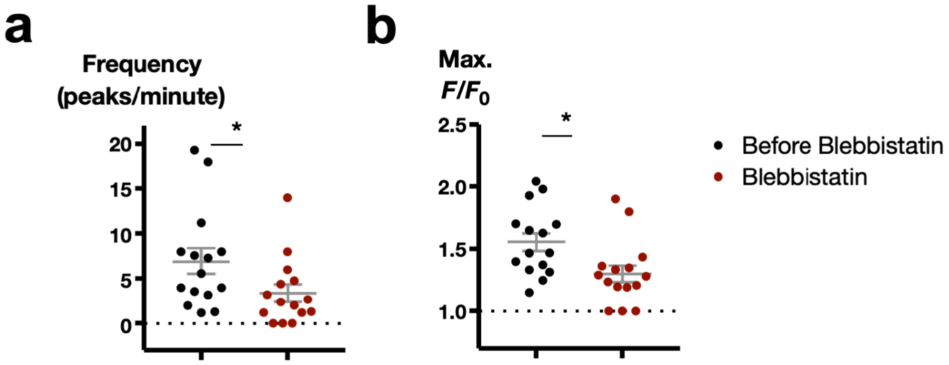

Supplementary Fig. 18. GenEPi responses decrease when cardiomyocytes contraction stops in response to blebbistatin. (a) Frequency of peaks, and (b) maximum F/F_0 measurement for each ROI before, and after the addition of 100 µM blebbistatin. n=15 ROIs, error bar: SEM. Two-tailed Wilcoxon rank-sum test. Source data are provided as a Source Data file.

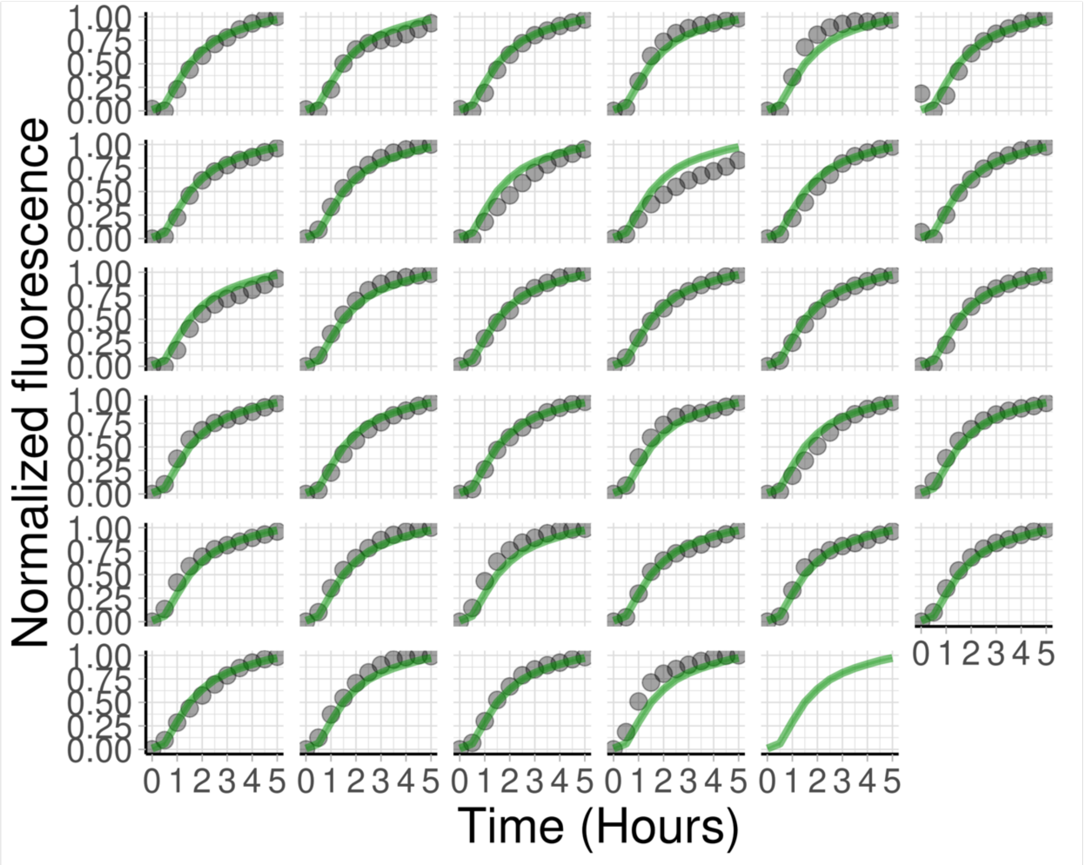

Supplementary Fig. 19. Longitudinal analysis of GenEPi expression post-heat-shock in *Tg(hsp70:GenEPi)* zebrafish. Each graph displays the normalized fluorescence intensity of 35 individual *Tg(hsp70:GenEPi)* zebrafish post-heat-shock presented in **Fig. 5c**. Data were plotted in PlotTwist (Shiny apps). Source data are provided as a Source Data file.

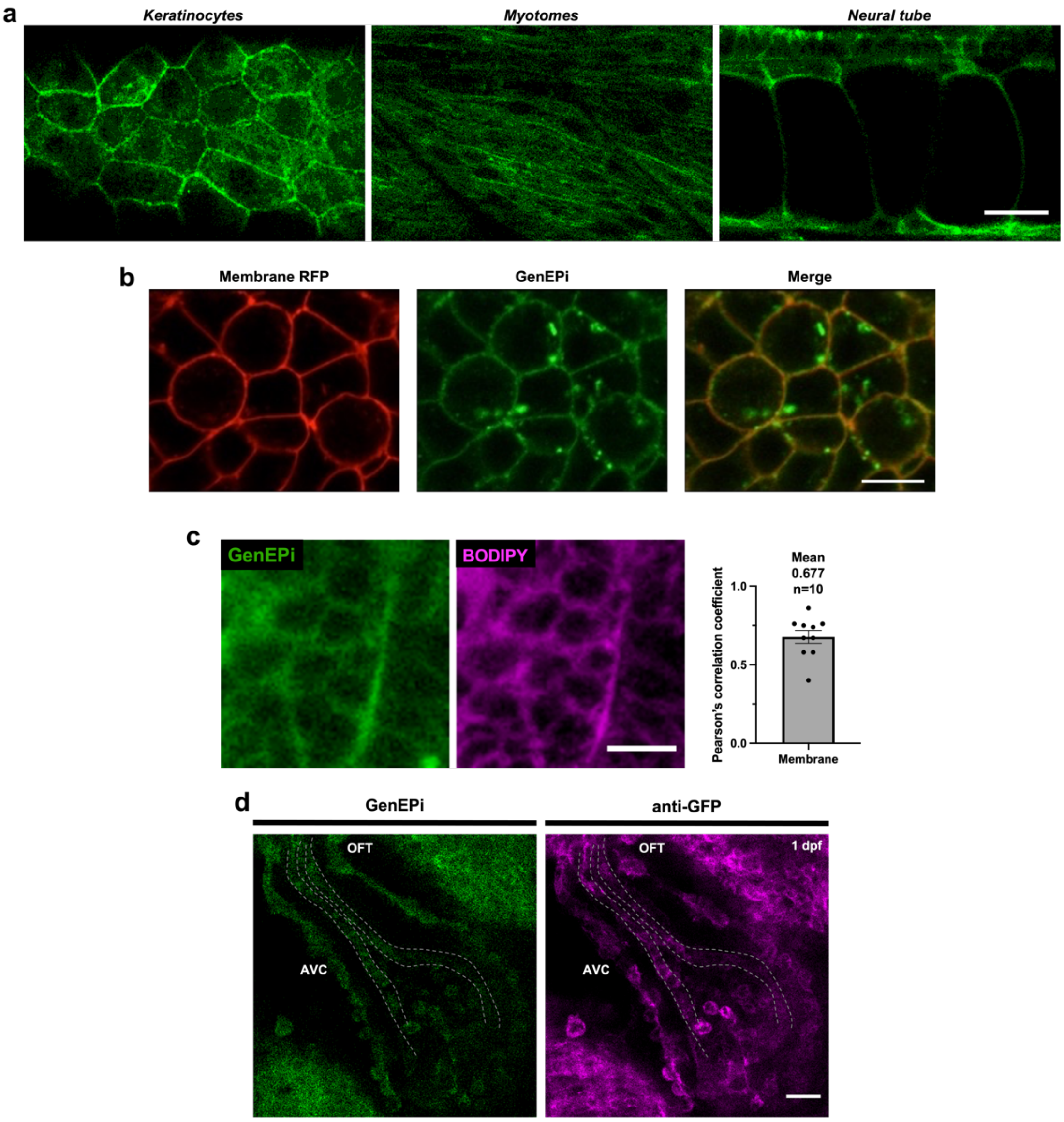

Supplementary Fig. 20. GenEPi is homogeneously expressed and mainly localizes to the plasma membrane of individual cells after heat-shock of *Tg(hsp70:GenEPi)* zebrafish. (a) Representative GenEPi expression in keratinocytes, myotomes and part of the neural tube in *Tg(hsp70:GenEPi)* zebrafish at 30 hpf. Scale bar, 20 µm. (b) GenEPi colocalizes with membrane-RFP in *Tg(hsp70:GenEPi)* zebrafish injected with membrane-RFP mRNA at 12 somites stage (14 hpf). Scale bar, 10 µm. (c) Colocalization analysis of GenEPi and BODIPY in *Tg(hsp70:GenEPi)* zebrafish at 30 hpf and Pearson's correlation coefficient. n=10 ROIs with at least 2 individual cells. Data are presented as means ± SEM. Scale bar, 10 µm. (d) Anti-GFP immunostaining of heat-shocked *Tg(hsp70:GenEPi)* zebrafish, which confirms the expression of GenEPi in endocardium and myocardium cells at 1 dpf. Scale bar, 20 µm. Source data are provided as a Source Data file.

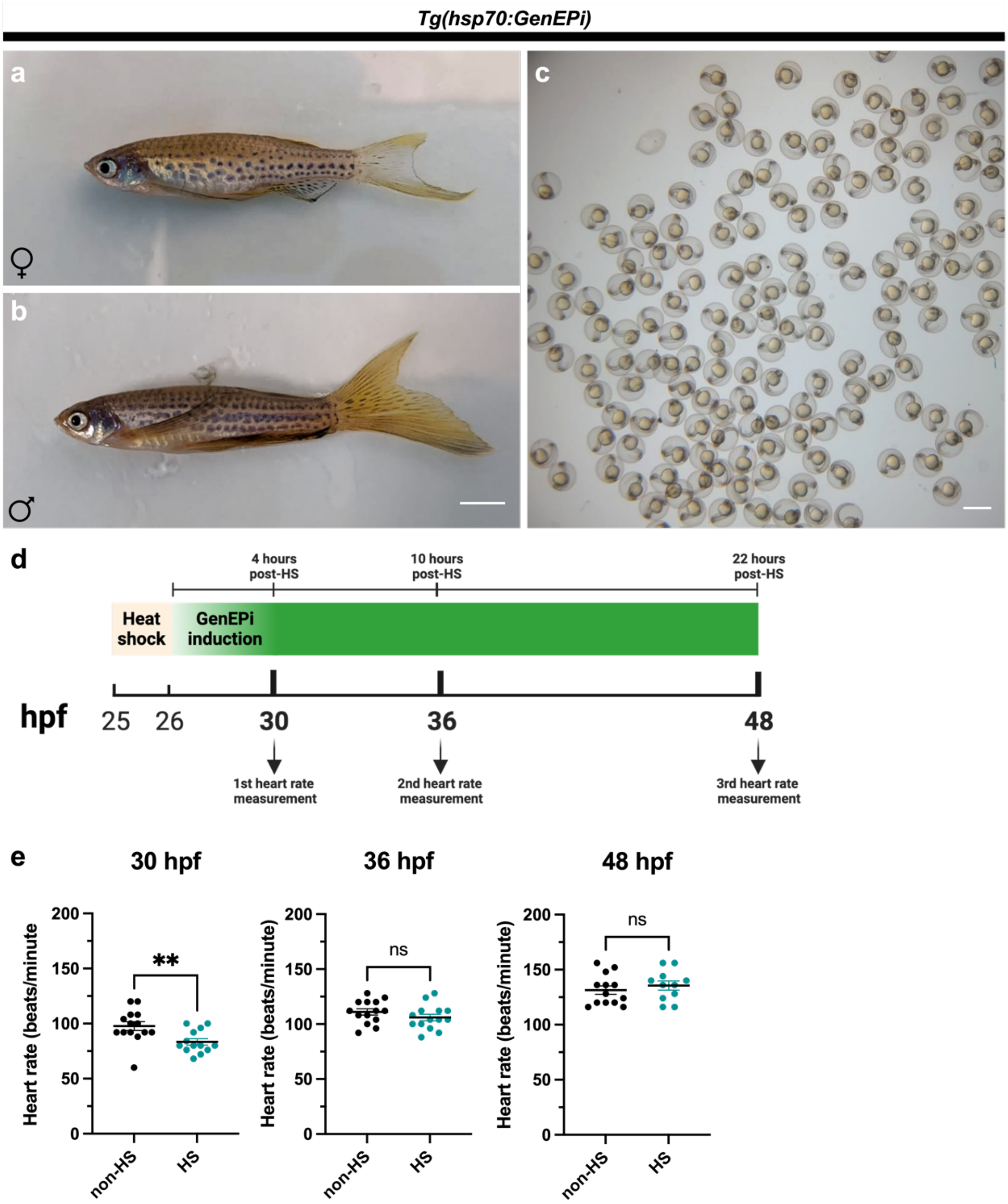

Supplementary Fig. 21. Transient induction of systemic expression of GenEPi in transgenic embryos does not affect their development to adulthood. (a) Brightfield image of a female and (b) male *Tg(hsp70:GenEPi)* adult zebrafish (WT/TL background) that were heat-shocked to induce GenEPi expression at 1 dpf. Scale bar, 5 mm. (c) *Tg(hsp70:GenEPi)* zebrafish produce healthy embryos when crossed. Scale bar, 1 mm. (d) Time course of the heart beating measurement experiments presented in (e). (e) Heart rate of individual *Tg(hsp70:GenEPi)* zebrafish heat-shocked (HS) to induce GenEPi expression at 1 dpf. Ordinary one-way ANOVA test. Data are presented as means ± SEM. Notably, the heart rate of the embryos does not differ to control non-heat-shocked (non-HS) zebrafish when the latter have recovered from the heat-shock process. Source data are provided as a Source Data file.

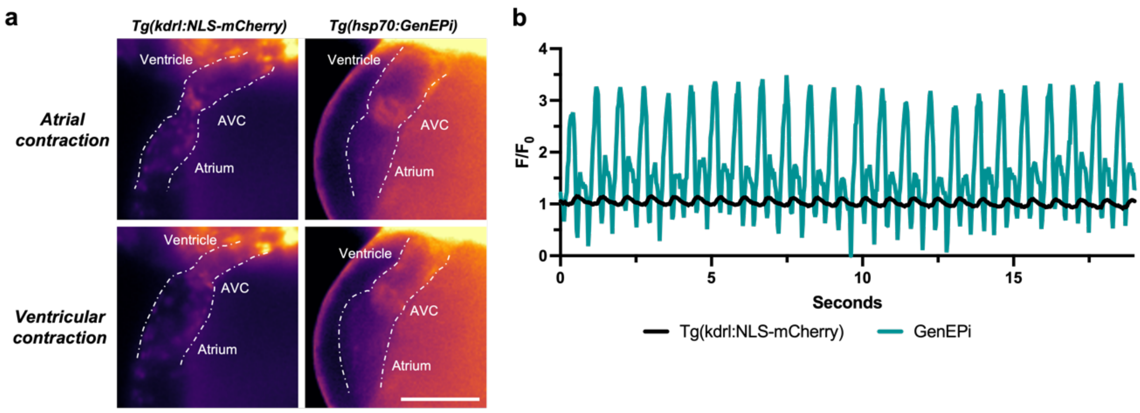

Supplementary Fig. 22. GenEPi response is different from the typical fluorophore motion artifact observed during zebrafish heart beating. (a) Representative images of a *Tg(kdrl:NLS-mCherry)* and *Tg(hsp70:GenEPi)* zebrafish hearts at 1dpf during atrial and ventricular contraction. AVC: atrioventricular canal. Scale bar, 100 µm. White dashed lines outline the zebrafish heart. (b) Representative intensity profiles (F/F_0) of the AVC in *Tg(kdrl:NLS-mCherry)* and *Tg(hsp70:GenEPi)* zebrafish hearts. Note that GenEPi shows a bi-phasic response, while the kdrl:NLS-mCherry signal shows the typical artifact increase due to cell motion of the heart contraction. Source data are provided as a Source Data file.

Supplementary Table 1. Indicative list of biological processes Piezo1 is involved in.

Biological processes in which Piezo1 is important	References
**Cardiovascular mechanosensation**	(Fotiou et al., 2015, Huang et al., 2016, Liang et al., 2017, Lukacs et al., 2015, Ploeg et al., 2021, Retailleau et al., 2015, Wang et al., 2016, Zhang et al., 2021)
**Erythrocyte mechanosensation and volume regulation**	(Albuisson et al., 2013, Andolfo et al., 2013, Bae et al., 2013, Nguetse et al., 2020, Zarychanski et al., 2012)
**Neural mechanosensation and differentiation**	(Blumenthal et al., 2014, Pathak et al., 2014, Velasco-Estevez et al., 2020)
**Epithelial cell mechanosensation**	(Eisenhoffer et al., 2012, Gudipaty et al., 2017, Kulkarni et al., 2021, Peyronnet et al., 2013)
**Bladder mechanosensation**	(Dalghi et al., 2021, Miyamoto et al., 2014)
**Kidney mechanosensation**	(Martins et al., 2016)
**Cartilage mechanosensation**	(Lee et al., 2014, Lee et al., 2021)
**Cell adhesion**	(Aglialoro et al., 2020, McHugh et al., 2010, McHugh et al., 2012)
**Cell migration**	(Canales Coutiño and Mayor, 2021, Holt et al., 2021, Hung et al., 2016, Li et al., 2015, Yang et al., 2014)
**Plant mechanosensation**	(Radin et al., 2021, Mousavi et al., 2021)

Supplementary Table 2. List of primers.

Oligo name	Oligo sequence (5'> 3')
AgeI-F (GCaMPs-G1-G5)	CCAACCGGTATGGGTTCTCATCATCATCATCA
1xGSGG-F (GCaMPs-G1-G5)	CCAACCGGTGGAGGATCCGGCATGGGTTCTCATCATCATCATCA
2xGSGG-F (GCaMPs-G1-G5)	CCAACCGGTGGATCAGGAGGTGGATCAGGAGGTATGGGTTCTC ATCATCATCATCA
GCaMP-R-NotI (GCaMPs-G2-G5)	TTTTCCTTTTGCGGCCGCTTTTTTCCTTCCTTACTTCGCTGTCATC ATTTGTACA
R-6s-EF4 (GCaMP-G1)	GCAGCCTAGGTATTAATCAATTAGT
hP1-F-HindIII	CCAAAGCTTATCGCATGGAGCCGCACGT
hP1-R-AgeI	TGAACCGGTCTCCTTCTCACGAGTCCACTTGATC
egfp-F	CCAACCGGTGGATCAGGAGGTGGATCAGGAGGTATGGTGAGCA AGGGCGAGG
egfp-R	TTTTCCTTTTGCGGCCGCTTTTTTCCTTCCTTAGGACTTGTACAGC TCGTCCAT
hP1-F-AgeI	CCAACCGGTATCGCATGGAGCCGCACGT
hP1-R-NotI	TTTTCCTTTTGCGGCCGCTTTTTTCCTTCCTTACTCCTTCTCACGA GTCCACTTGATC
gBlock containing Lck targeting sequence	GGACTCAGATCTGCCACCATGGGCTGTGGCTGCAGCTCAAACC CTGAAGATGACTGGATGGAGAACATTGACGTGTGTGAGAACTGC CATTATCCCCCGCCGAAGCTTCGAATTGATCCACCGGATCTCGC CACCACCGGTCCA

Oligo name	Oligo sequence (5'>3')
Gibson Assembly F	GAAGTAAGGAAGGAAAAAAGCGGCCGCGTCTAGAGATCCAGACATGATAAGATACAT TG
Gibson Assembly R	CATCATTTTGGCAAAGAAATCAAGCTTATCGCATGGAGCCGCACGT
GenEPiSeqFW1	GAGGTCTATATAAGCAGAGCTGG
GenEPiSeqFW2	CTACCTGCTGCTCTTCCTGG
GenEPiSeqFW3	CTGTATGGGATGACGCTGTG
GenEPiSeqFW4	CTCACAGGCAGGATGCAGTG
GenEPiSeqFW5	CAGCTGGACCAGGATCTGC
GenEPiSeqFW6	CGTCACCGTCATCATCTCCAA
GenEPiSeqFW7	GAACAGGCAGGACAGCTACC
GenEPiSeqFW8	ATCCCGAGGCCCAGCAA
GenEPiSeqFW9	ATCATCATTTTTGGCTTCTGGGC
GenEPiSeqFW10	CTGTTCACCATGAGCGCC
GenEPiSeqFW11	GACCGCATCCTCAAGCTCT
GenEPiSeqFW12	GGCAAGCTGACCCTGAAGTT
XLGenEPi_S217L_FW	TGGCCCTCTCCAGTGTCTACCTGCTGCTC
XLGenEPi_S217L_R	GGGGGTGGGCGATGCCTGCCAGTGCAAG

Supplementary Table 3. Statistical data for the systematic reporter screen depicted in Figure 1c.

sample sizes (n)					
sample	cytosolic	no linker	1xGSGG	2xGSGG	stimuli
GCaMP G1	20	15	15	13	shear stress
	18	15	15	9	ionomycin
GCaMP G2	18	13	18	14	shear stress
	21	19	16	16	ionomycin
GCaMP-G3	17	9	15	14	shear stress
	15	17	16	15	ionomycin
GCaMP-G4	13	14	12	17	shear stress
	18	13	15	12	ionomycin
GCaMP-G5	18	14	11	14	shear stress
	19	11	14	17	ionomycin
eGFP			16		shear stress
			19		ionomycin

p value (Shapiro-Wilk normality test)					
sample	cytosolic	no linker	1xGSGG	2xGSGG	stimuli
GCaMP-G1	0.4059	0.8072	0.0014	0.0424	shear stress
	0.0507	0.0004	0.0106	0.4371	ionomycin
GCaMP-G2	0.2206	0.498	0.7162	0.681	shear stress
	0.0787	0.3892	0.0018	0.3099	ionomycin
GCaMP-G3	<0.0001	0.0139	0.0751	0.2232	shear stress
	0.2435	<0.0001	0.0171	0.4208	ionomycin
GCaMP-G4	0.796	0.6878	0.0384	0.1161	shear stress
	0.0415	0.2133	0.0002	0.0069	ionomycin
GCaMP-G5	0.0406	0.9933	0.223	0.2232	shear stress
	0.0183	0.0123	0.0879	0.001	ionomycin
eGFP			0.0263		shear stress
			0.1312		ionomycin

shear stress response (F/F0)				
**p value**	no linker	1xGSGG	2xGSGG	sample
cytosolic vs.	0.4232	0.0427	0.1694	GCaMP-G1
cytosolic vs.	0.3668	0.0035	0.0515	GCaMP-G2
cytosolic vs.	0.0388	0.0078	0.0209	GCaMP-G3
cytosolic vs.	0.7339	0.0188	0.0432	GCaMP-G4
cytosolic vs.	0.0001	0.0922	0.0143	GCaMP-G5

ionomycin response (F/F0)				
**p value**	no linker	1xGSGG	2xGSGG	sample
cytosolic vs.	<0.0001	<0.0001	0.0016	GCaMP-G1
cytosolic vs.	0.0002	0.0004	<0.0001	GCaMP-G2
cytosolic vs.	0.0003	<0.0001	0.0003	GCaMP-G3
cytosolic vs.	0.0036	<0.0001	0.3684	GCaMP-G4
cytosolic vs.	0.0183	0.0001	0.0231	GCaMP-G5

shear stress vs. ionomycin response (F/F0)				
sample	no linker	1xGSGG	2xGSGG	
GCaMP-G1	<0.0001	0.766	0.0004	**p value**
GCaMP-G2	0.5842	0.2811	0.53	
GCaMP-G3	0.7916	0.6189	0.0024	
GCaMP-G4	0.011	<0.0001	0.4187	
GCaMP-G5	0.2441	<0.0001	0.0586	

F/F0		
**p value**	PiezoG4	
Piezo1-eGFP vs.	<0.0001	shear stress (10 dyne/cm2)
	0.9454	ionomycin (1 µM)
