# Supplementary material for: Highly specific and non-invasive imaging of Piezo1-dependent activity across scales using GenEPi
Source: Nat Commun. 2023 Jul 19;14:4352. doi: 10.1038/s41467-023-40134-y (PMC10356793; doi:10.1038/s41467-023-40134-y)
Supplement: Supplementary file 2 — Description of Additional Supplementary Files [file 41467_2023_40134_MOESM2_ESM.pdf]

File Name: Supplementary Movie 1
Description: TIRFM imaging of a GenEPi expressing HEK293T cell. Video of a representative HEK293T cell transiently expressing GenEPi. Time stamp in seconds, and scale bar 5 µm.

File Name: Supplementary Movie 2
Description: Spontaneous beating of differentiated GenEPi cardiomyocytes. Video of a representative spontaneously beating differentiated cardiomyocyte patch. Time stamp in seconds, and scale bar 20 µm.

File Name: Supplementary Movie 3
Description: GenEPi-expressing cells response to beating cardiomyocytes in a differentiated microtissue. Video of representative GenEPi expressing cells within a non-dissected spontaneously beating cardiomyocyte patch. Time stamp in seconds, and scale bar 5 µm.

File Name: Supplementary Movie 4
Description: Live imaging of Tg(hsp70:GenEPi) zebrafish heart. Representative video of heart beating in a heatshocked Tg(hsp70:GenEPi) zebrafish. Scale bar 100 µm.
